# Association of Dietary Live Microbe Intake with Cardiovascular Disease in US Adults: A Cross-Sectional Study of NHANES 2007–2018

**DOI:** 10.3390/nu14224908

**Published:** 2022-11-20

**Authors:** Lu Han, Qi Wang

**Affiliations:** 1 Department of Obstetrics and Gynecology, First Affiliated Hospital, Xi’an Jiaotong University, Xi’an 710061, China; 2 Department of Gynecologic Oncology, Shaanxi Provincial Cancer Hospital, Xi’an 710061, China

**Keywords:** dietary live microbe, cardiovascular diseases, stroke, congestive heart failure, cross-sectional study

## Abstract

Objective: To detect the potential association between dietary live microbe and cardiovascular diseases (CVD). Methods: Data of 10,875 participants aged 18 years or older in this study were collected from the National Health and Nutrition Examination Survey (NHANES). Participants in this study were divided into three groups according to the Sanders dietary live microbe classification system: low, medium, and high dietary live microbe groups. CVD was defined by a combination of self-reported physician diagnoses and standardized medical status questionnaires. The analyses utilized weighted logistic regression models. Results: After the full adjustment for confounders, patients in the medium dietary live microbe group had a low prevalence of CVD in contrast to those in the low dietary live microbe group (OR: 0.78, 95% CI: 0.52–0.99, and *p* < 0.05), but no significant association with CVD was detected between the high and low dietary live microbe groups. Higher dietary live microbe groups were negatively associated with the prevalence of stroke (*p* for trend = 0.01) and heart attack (*p* for trend = 0.01). People who were male were more likely to suffer stroke due to low dietary live microbe (*p* for interaction = 0.03). Conclusion: A high dietary live microbe intake was associated with a low prevalence of CVD, and the significant association was detected when the analysis was limited to stroke and heart attack.

## 1. Introduction

Although more hygienic food has clear positive effects on public health, the reduction in microbial exposure may also have unanticipated adverse health effects. The potential for a microbial role is addressed in “the old friends hypothesis” [1], which suggests that an important and beneficial source of immune stimulation is exposure to nonharmful or commensal microbes [2]. Several studies in the past few decades have demonstrated the beneficial effects of dietary microorganisms on human health [3]. Live safe microbes obtained from daily intake in the diet may reach the gut and merge with the resident microbiota, improving intestinal function, regulating the immune system, and minimizing susceptibility to chronic diseases [4].

Cardiovascular disease (CVD) is one of the leading causes of death for both men and women. According to the report from the American Heart Association and the National Institutes of Health, cardiovascular has become the single most affected system, leading to the highest number of deaths in the USA and worldwide [5]. The global number of deaths from CVD has increased during the past decade by 12.5%, and now accounts for approximately one third of all deaths globally [6].

The relationship between cardiovascular disease and the consumption of living microbes in the diet has not been explored explicitly. Fermented dairy and other products were confirmed have an association with the incidence of cardiovascular disease [7,8,9,10,11]. However, not only fermented foods include living microbes, but also a wide variety of other foods, including raw, unpeeled fruits and vegetables [12,13]. Sanders et al. have assessed the number of live microbes that are consumed in the diet and categorized all foods into low (<10^4^ CFU/g), medium (10^4^–10^7^ CFU/g), or high (>10^7^ CFU/g) levels of live microbes.

Based on Sanders’ dietary live microbe’ classification system, an association of dietary live microbes and cardiovascular diseases was conducted based on surveillance data from the National Health and Nutrition Examination Survey (NHANES) 2007–2018.

## 2. Methods

### 2.1. Data Source and Participants

**Figure 1:**
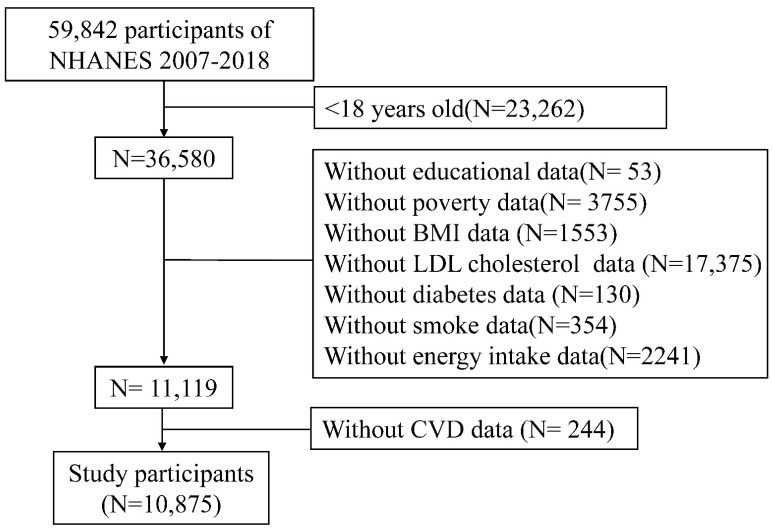
Flow chart of the study.

The National Center for Health Statistics (NCHS, Hyattsville, MD, USA), CDC conducts the NHANES, a large, multistage, complex survey of civilian, noninstitutionalized US populations. A representative sample of the US population was sampled using a stratified, multistage cluster probability sampling design. For this analysis, six cycles of NHANES (2007–2008, 2009–2010, 2011–2012, 2013–2014, 2015–2016, 2017–2018) with independent samples were utilized to accumulate an appropriate sample size. Individuals were excluded if they were under the age of 18 or if they provided missing values for important variables. The inclusion and exclusion criteria in this study are summarized in the flowchart (Figure 1). There were no differences in the total number of individuals with missing variables for each study year (Figure S1). The National Center for Health Statistics and Research’s Ethics Review Committee has approved the NHANES studies. The consent form was signed by every participant in the survey. The NHANES database is accessible without ethical or administrative approval.

### 2.2. Dietary Intakes and Live Microbial Category

The National Center for Health Statistics linked the 24-h eating data to the US Department of Agriculture Food Surveys Nutrient Database to estimate energy and nutrient intake. According to Sanders’ research, the method to estimate quantities of live microbes (per gram) for 9388 food codes contained in 48 subgroups in the NHANES database. The foods with different live microorganisms were categorized as low (10^4^ CFU/g), medium (10^4^–10^7^ CFU/g), or high (>10^7^ CFU/g) by four experts in the field. For these assessments, four experts relied on consulting the literature, authoritative reviews, and known effects of food processing (for example, pasteurization) on microbial viability. The differences were solved by reconciling within and between the teams, and external consulting with Fred Breidt, USDA Agricultural Research Service Microbiologist [14].

### 2.3. Outcome Definitions

CVD was determined during individual interviews using self-reported physician diagnoses and standardized medical status questionnaires. The participants were asked, “Has a doctor or other health professional ever told you that you have congestive heart failure (CHF)/coronary heart disease (CHD)/angina pectoris/heart attack/stroke?”. Participants who answered “yes” to any of the above questions were considered CVD patients. The outcome was thus converted to a dichotomous variable.

### 2.4. Covariates

The following covariates based on evidence of associations with dietary live microbes and CVD based on both clinical experience and the existing literature. Covariates included age [15]; sex (men/women) [15]; race (Mexican American, Other Hispanic, Non-Hispanic White, Non-Hispanic Black, Other Races) [15]; family income-to-poverty ratio(FIR)(<1.2, or ≥1.2); educational level (<high school, high school, or >high school) [15]; energy intake (kcal/day) [16]; -serum low density lipoprotein cholesterol (ldl-cholesterol) (mg/dL) (mg/dL) [17]; physical activity (vigorous/moderate recreational activities for at least 10 min continuously in a typical week) [18]; smoke [19]; BMI(normal weight, overweight, obesity); hypertension [20]; and diabetes mellitus [21]. BMI was calculated as weight (kg) divided by height (meters squared), which was divided into normal weight (BMI < 25), overweight (25 ≤ BMI < 30), and obesity (BMI ≥ 30). Fasting serum samples were collected by venipuncture to measure fasting glucose, hemoglobin A1c (HbA1c), and ldl-cholesterol. The participants consumed 75 g of dextrose as soon as their fasting specimen was collected, followed by the oral glucose tolerance test (OGTT) 2 h later. The mean value was calculated based on three measurements of diastolic blood pressure (mmHg) and systolic blood pressure (mmHg) taken by each participant. Participants were considered to have hypertension for any of the following reasons: systolic blood pressure ≥140 mm Hg and/or diastolic blood pressure ≥90 mm Hg, any self-reported diagnosis of hypertension, or any self-reported use of antihypertensive drug [22]. Participants were considered to have diabetes for any of the following reasons: HbA1c ≥ 6.5%, fasting glucose ≥126mg/dL, serum glucose at 2 h following a 75 g glucose load (OGTT) ≥200mg/dL, any self-reported diagnosis of diabetes, or any self-reported use of insulin or other diabetes medication [23].

### 2.5. Statistical Analyses

An appropriate NHANES sample weight was applied for the complex multistage cluster design. Continuous variables were displayed as mean ± standard deviation (SD). Categorical variables were displayed as numbers (weighted percentage). Comparative analysis of categorical variables was conducted using the Mann–Whitney U test and the Mann–Whitney U test for continuous variables. Participants in this study were divided into three groups according to the food code assignments of low, medium, and high: Low dietary live microbe group (all foods are Lo); Medium dietary live microbe group (any foods are Med but not Hi); High dietary live microbe group (any foods are Hi). Survey-weighted multiple logistic regression analysis was used to evaluate the relationships between dietary live microbe intake and the prevalence of CVD, and three models were constructed. Model 1 was adjusted for age and gender. Model 2 was adjusted for the Model 1 variables plus other demographic factors (race, BMI, FIR, and education). Model 3 was adjusted for the Model 2 variables plus smoking, physical activity, diabetes, hypertension, serum ldl-cholesterol, and energy intake. The subgroup analysis was performed using multivariate logistic regression stratifying by age, sex, race, education level, FIR, cycle year, BMI, smoking status, vigorous activity, diabetes, and hypertension. An interaction term was also used to explore the heterogeneity of the association between different subgroups using the log-likelihood ratio test model. R software version 4.1.2. (Core Team, Vienna, Austria) was used to conduct all the statistical analyses. *p* value <0.05 was considered statistically significant.

## 3. Results

### 3.1. Characteristics of the Included Population

Our study included 10,875 participants, and the overall weighted prevalence of CVD was 10.98%, 9.75%, and 7.94% being in the low, medium, and high dietary live microbe groups, respectively. Table 1 displays the baseline characteristics of the study population. Participants with CVD were more likely to be older, male, Non-Hispanic White, equal to or less than high school, less energy intake, less activity, higher blood ldl-cholesterol levels, smokers, obese, and have hypertension or diabetes. Furthermore, participants with CVD were more likely to consume fewer dietary live microbes (*p* = 0.01).

### 3.2. Subjects in Different Dietary Live Microbe Groups

All participants were divided by food code assignments of low, medium, and high. The clinical characteristics of the three groups are shown in Table 2. Participants in the high vs. low dietary live microbe group were older and more likely to be female; less activity; smoking; Non-Hispanic Black; high education levels; less smoking; normal weight; more energy intake; high FIR; and without hypertension or diabetes (*p* < 0.05).

### 3.3. Association between Different Dietary Live Microbe Groups and CVD

Results of univariable and multivariable weighted logistic regression analyses of different dietary live microbe groups and CVD are displayed in Table 3. As is shown, in the univariate logistic regression analysis, participants in high dietary live microbe group had a low prevalence of CVD in contrast to those in the low dietary live microbe group (odds ratio (OR): 0.70, 95% confidence interval (CI): 0.55–0.88, and *p* < 0.05). There was no significant association between the medium dietary live microbe group and CVD compared with the low dietary live microbe group. In model 1 (adjusted for age and gender) and model 2(adjusted for model 1 plus Race, BMI, FIR, and educational level), the prevalence of CVD was significantly lower in the high and medium dietary live microbe groups respectively, when compared with those of the low dietary live microbe group (*p* for trend <0.05). Through adjustment for model 3 (model 2 plus smoking, physical activity, diabetes, hypertension, serum ldl-cholesterol, and energy intake), it became clear that patients in the medium dietary live microbe group had a low prevalence of CVD in contrast to those low dietary live microbe group (OR: 0.78, 95% CI: 0.62–0.99, and *p* < 0.05), but no significant association with CVD was detected between the high and the low dietary live microbe groups.

### 3.4. Association between Different Dietary Live Microbe Groups and Subtypes of CVD

**Figure 2:**
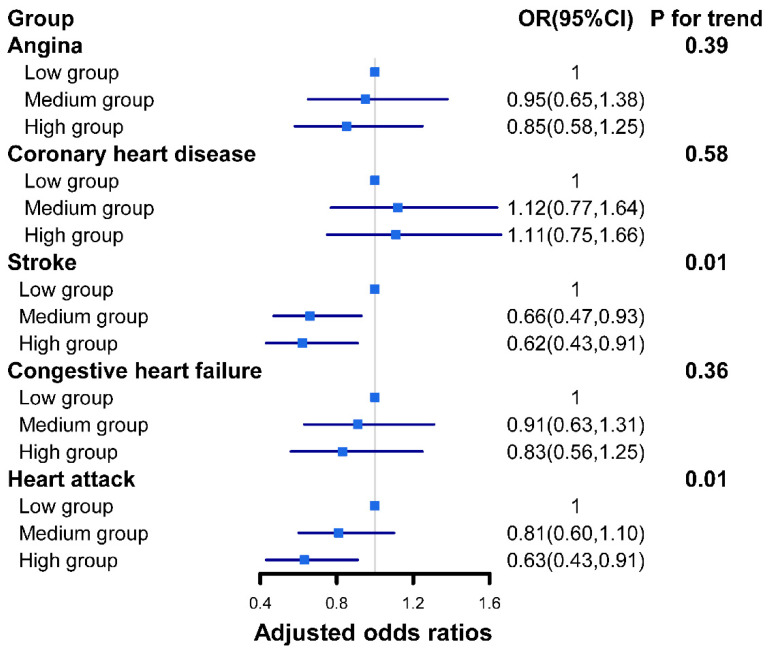
Adjusted odds ratios of cardiovascular disease subtypes with different dietary live microbe group in US adults in 2007–2018. The model is adjusted for age, gender, race, BMI, FIR, education, smoking, physical activity, diabetes, hypertension, blood ldl-cholesterol, and energy intake. OR, odds ratio; CI, confidence interval.

Figure 2 shows the results of the associations between different dietary live microbe groups and subtypes of CVD. Higher dietary live microbe groups were negatively associated with the prevalence of stroke (*p* for trend = 0.01) and heart attack (*p* for trend =0.01), but not with angina (*p* for trend = 0.39), congestive heart failure (*p* for trend = 0.0.36), or coronary heart disease (*p* for trend = 0.58). Compared with the low dietary live microbe group, the adjusted ORs (95% CIs) in the high dietary live microbe group were 0.62(0.43–0.91) and 0.63 (0.43–0.91) for stroke and heart attack, respectively. In addition, patients in the medium dietary live microbe group had a lower prevalence of stroke in contrast to those the low dietary live microbe group (OR: 0.66, 95% CI: 0.47–0.93, and *p* < 0.05).

### 3.5. Subgroup Analyses

The results of the subgroup analyses can be seen in Table 4. Compared with participants in the low dietary live microbe group, people who were male, who were non-Hispanic white, who have higher income, who never engaged in physical activity, who were obese, who were non-smokers, who were without hypertension, and with diabetes mellitus had a lower prevalence of stroke in the high dietary live microbe group (*p* = <0.01, 0.03, 0.03, 0.02, 0.04, 0.03, 0.04 and 0.01, respectively). Statistically, there was a significant difference between males and females, and the *p* for interaction was 0.03. Compared with participants in the low dietary live microbe group, people who were female, who were older than 60, who were non-Hispanic white, who never engaged in physical activity, who were obese, who were former smokers, and with diabetes mellitus had a lower prevalence of heart attack in the high dietary live microbe group (*p* = 0.04, 0.02, 0.04, 0.02, 0.04, 0.01, 0.01, and 0.01, respectively). However, no significant interaction was found for all above predefined factors.

## 4. Discussion

In this study, we found that the high dietary live microbes were negatively associated with the prevalence of all CVD and that the significant association existed when the analysis was limited to stroke and heart attack. Significantly, people who were male were more likely to suffer stroke due to low dietary live microbe.

The amounts and types of microbes in food including bacteria, yeasts, and molds depend on the type of food, its origin, and its processing level. The microbes of fresh fruits and vegetables show a wide range, but are often <10^6^ CFU/g [12], and fermented foods (e.g., yogurt) may contain 10^8^–10^11^ CFU/g [24]. Probiotics are defined as “live microorganisms that, when administered in adequate amounts, confer a health benefit on the host” in 2012 by the Food and Agriculture Organization of the United Nations (FAO)/World Health Organization (WHO), which was gradually modified and eventually widely accepted [19]. The human diet is made up of a wide variety of products that are sources of probiotic strains, including yogurt, kefir, sauerkraut, tempeh, and kimchi. Bacterial genera (Bifidobacterium, Bacillus, Enterococcus, Escherichia coli, Lactobacillus, Lactococcus, Leuconostoc, Pediococcus, Propionibacterium, Streptococcus) and yeast (Saccharomyces) are all known probiotics [25]. The mechanism of association between the high dietary live microbes and CVD may be as following reasons. First, the increased production of short chain fatty acids (butyrate, formate, propionate, and acetate) caused by probiotics has positive effects, including maintenance of gut integrity, regulating metabolism, cardiovascular, and inflammatory biomarkers [25]. Second, a number of probiotics, including Bifidobacterium, L acidophilus, and Bifidum, may reduce elevated cholesterol levels and support the prevention and treatment of some cardiovascular diseases [26,27]. Third, probiotics such as L. fermentum and L. reuteri could reduce oxidative stress, pro-inflammatory cytokines, and inflammation, which contribute to atherosclerosis pathogenesis and progression [28,29,30].

According to this article’s research findings, there are benefits to eating foods that provide dietary live microbe intake for adults in order to prevent cardiovascular diseases. However, probiotics are not appropriate for everyone and are contraindicated in several patient groups (cancer, auto-immune disease, transplantation, the elderly). Despite their potential benefits, probiotics may cause diarrhea in immunocompromised people. Moreover, it is possible that probiotics can cause sepsis in rare cases [31]. A more detailed investigation is needed to determine if probiotics are beneficial for cancer patients, patients with autoimmunity diseases, transplant patients, and the elderly.

As far as we know, this is the first study about the association between dietary live microbes, but not certain fermented foods or probiotics, and the prevalence of CVD in a large representative US population. The data used in this study came from the NHANES, a nationally representative database in the United States, and all of the data we used was subjected to rigorous quality control to ensure their validity. However, there are still some limitations of the current study. First, the self-reported diagnosis of CVD resulted in an information bias in the data collected on CVD outcomes. Second, dietary live microbes were categorized by experts’ discussion, consulting the literature or authoritative reviews, and consulting with microbiologist. Direct detection or cultivation may obtain more precise results, but this needs long time and huge expenditure. Third, participants were elementarily divided into the low dietary live microbe group (all foods are low live microorganism category); the medium dietary live microbe group (any food is medium live microorganism category but not high); and the high dietary live microbe group (any food is live microorganism category). This simple classification without precise calculation has a disposition to cause errors, and the exact test and calculation of daily dietary live microorganism need to be further explored. Fourth, cross-sectional measurements, but not follow-up measurements, are incapable of establishing temporal, let alone causative, relationships between the factors being evaluated, which might result in opaque recommendations or misinterpretations. Fifth, our research conclusion only applies to Americans, and cannot be extended to other regions due to eating behavior changes. Last, even though as many confounding variables as possible were taken into account, such as demographic characteristics, way of life, diet, and certain diseases, some confounding may still affect the results.

## 5. Conclusions

In conclusion, a high dietary live microbe intake was associated with a low prevalence of CVD, and the significant association was detected when the analysis was limited to stroke and heart attack, but causative relationship needs further RCT research.

## Figures and Tables

**Table 1:** The clinical characteristics of the study population with and without CVD.

Variable	CVD		*p*-Value
	No	Yes	
No. of participants	9616	1269	
Age [years]	47.05 (0.30)	65.39 (0.52)	<0.0001
Energy intake [kcal]	4189.88 (23.69)	3823.38 (64.73)	<0.0001
Serum ldl-cholesterol [mg/dL]	99.72 (1.43)	116.08 (0.58)	<0.0001
Gender (%)			<0.001
Female	5164 (53.34)	555 (46.54)	
Male	4442 (46.66)	714(53.46)	
Race/ethnicity (%)			<0.0001
Mexican American	1445 (7.95)	107 (4.58)	
Non-Hispanic Black	1886 (10.02)	268 (11.38)	
Non-Hispanic White	4191 (68.83)	722 (75.40)	
Other Hispanic	979 (5.62)	103 (3.25)	
Other Racer	1105 (7.58)	69 (5.40)	
FIR (%)			0.03
<1.2	2590 (18.78)	383 (22.05)	
≥1.2	7016 (81.22)	7016 (81.22)	
Educational status (%)			<0.0001
>high school	5460 (63.91)	547 (49.37)	
high school	2122 (22.54)	333 (27.34)	
<high school	2024 (13.55)	389 (23.29)	
Vigorous/moderate recreational activities (%)			<0.0001
Yes	4870 (55.66)	422 (39.86)	
No	4736 (44.34)	847 (60.14)	
Smoke (%)			
Now	1803 (18.14)	266 (20.89)	<0.0001
Former	2261 (25.13)	485 (40.73)	
Never	5542 (56.73)	518 (38.38)	
Body Mass Index (%)			<0.0001
Normal	2827 (30.41)	273 (22.95))	
Overweight	3159 (32.99)	400 (29.17)	
Obesity	3620 (36.60)	596 (47.88)	
Hypertension (%)			<0.0001
Yes	3734 (35.97)	1008 (75.74)	
No	5872 (64.03)	261 (24.26)	
Diabetes mellitus (%)			<0.0001
Yes	1752 (14.14)	579 (40.47)	
Borderline	1681 (17.38)	237 (18.72)	
No	6173 (68.48)	453 (40.81)	
Dietary livemicrobe group (%)			0.01
Low	3380 (31.67)	515 (36.71)	
Medium	3910 (38.54)	503 (39.15)	
High	2316 (29.78)	251(24.14)	

CVD, Cardiovascular diseases; FIR, family income-to-poverty ratio.

**Table 2:** The clinical characteristics of the study population according to the different dietary live microbes.

Variable	Low Dietary Live Microbe Group	Medium Dietary Live Microbe Group	High Dietary Live Microbe Group	*p*-Value
No. of participants	3805	4413	2567	
Age [years]	46.71 (0.45)	50.43 (0.44)	48.99 (0.46)	<0.0001
Energy intake [kcal]	4003.75 (43.68)	4167.64 (37.62)	4303.42 (47.05)	<0.0001
Serum ldl-cholesterol [mg/dL]	114.53 (0.95)	113.84 (0.82)	115.35 (1.11)	0.51
Gender (%)				<0.001
Female	1906 (47.97)	2362 (54.35)	1451 (55.69)	
Male	1989 (52.03)	2051 (45.65)	1116 (44.31)	
Race/ethnicity (%)				<0.0001
Mexican American	469 (6.86)	786 (9.87)	297 (5.52)	
Non-Hispanic Black	1063 (15.07)	771 (9.43)	320 (5.68)	
Non-Hispanic White	1569 (64.55)	1935 (67.40)	1409 (77.59)	
Other Hispanic	366 (5.48)	454 (5.82)	262 (4.73)	
Other Racer	428 (8.03)	467 (7.49)	279 (6.48)	
FIR (%)				<0.0001
<1.2	1315 (27.00)	1120 (16.80)	538 (13.42)	
≥1.2	2580 (73.00)	3293 (83.20)	2029 (86.58)	
Educational status (%)				<0.0001
>high school	1867 (53.44)	2418 (62.94)	1722 (71.94)	
high school	995 (27.22)	979 (22.08))	481 (19.58)	
<high school	1033 (19.34)	1016 (14.99)	364 (8.48)	
Vigorous/moderate recreational activities (%)				<0.0001
Yes	1657 (45.49)	2196 (55.88)	1439 (61.37)	
No	2238 (54.51)	2217 (44.12)	1128 (38.63)	
Smoke (%)				<0.0001
Now	985 (26.77)	707 (14.92)	377 (13.79)	
Former	896 (23.57)	1176 (28.22)	674 (27.89)	
Never	2014 (49.66)	2530 (56.85)	1516 (58.32)	
Body Mass Index (%)				<0.0001
Normal	1043 (26.23)	1268 (30.38)	789 (32.59)	
Overweight	1214 (30.95)	1489 (32.87)	856 (34.13)	
Obesity	1638 (42.82)	1656 (36.75)	922 (33.28)	
Hypertension (%)				<0.001
Yes	1765 (41.53)	1951 (41.02)	1026 (36.26)	
No	2130 (58.47)	2462 (58.98)	1541 (63.74)	
Diabetes mellitus (%)				<0.0001
Yes	845 (16.92)	1019 (18.67)	467 (13.77)	
Borderline	706 (18.17)	792 (17.89)	420 (16.28)	
No	2344 (64.90)	2602 (63.44)	1680 (69.96)	
CVD (%)				0.01
Yes	515 (10.98)	503 (9.75)	251 (7.94)	
No	3380 (89.02)	3910 (90.25)	2316 (92.06)	

CVD, Cardiovascular diseases; FIR, family income-to-poverty ratio.

**Table 3:** Association between different dietary live microbe group and CVD.

Outcomes	Model	Low Dietary Live Microbe Group OR (95%)	Medium Dietary Live Microbe Group OR (95%)	High Dietary Live Microbe Group OR (95%)	*p* for Trend
**CVD**	Crude	1.00 (Reference)	0.88(0.72,1.06)	0.70(0.55,0.88) *	0.003
	Model1	1.00 (Reference)	0.67(0.53,0.84) *	0.61(0.48,0.79) *	<0.001
	Model2	1.00 (Reference)	0.77(0.61,0.97) *	0.76(0.59,0.99) *	0.039
	Model3	1.00 (Reference)	0.78(0.62,0.99) *	0.83(0.64,1.08)	0.148

Crude was univariate logistic regression. Model 1 was adjusted for age and gender. Model 2 was adjusted for age, gender, race, BMI, FIR, and educational level. Model 3 was adjusted for age, gender, race, BMI, FIR, education, smoking, physical activity, diabetes, hypertension, serum ldl-cholesterol, and energy intake. * *p* value < 0.05; CVD, Cardiovascular diseases.

**Table 4:** Subgroup analyses on the association between high vs. low dietary live microbe group with stroke and heart attack.

Subgroup Variable	Total Number = 6372, Stroke = 450			Total Number = 6372, Heart Attack = 438		
	OR High vs. Low (95% CI)	*p*-Value	*p*-Interaction	OR High vs. Low (95% CI)	*p*-Value	*p*-Interaction
Gender			0.03			0.93
Female	1.07 (0.65,1.78)	0.41		0.63 (0.41,0.97)	0.04	
Male	0.42 (0.25,0.73)	<0.01		0.74 (0.43,1.29)	0.29	
Age			0.05			0.81
<60	0.42 (0.18,0.99)	0.05		0.95 (0.44, 2.04)	0.89	
≥60	0.77 (0.54,1.12)	0.17		0.59 (0.37,0.93)	0.02	
Race/ethnicity			0.93			0.59
Mexican American	0.32 (0.10, 1.06)	0.06		0.86 (0.21, 3.60)	0.83	
Non-Hispanic Black	0.75 (0.31,1.81)	0.51		0.85 (0.30, 2.45)	0.76	
Non-Hispanic White	0.59 (0.37,0.94)	0.03		0.65 (0.44,0.98)	0.04	
Other Hispanic	1.21 (0.30, 4.92)	0.78		0.72 (0.15, 3.53)	0.68	
Other Racer	0.70 (0.27, 1.83)	0.46		0.54 (0.10, 2.96)	0.47	
FIR			0.83			0.33
<1.2	0.57 (0.21,1.52)	0.25		0.62 (0.41,0.92)	0.02	
≥1.2	0.63 (0.41,0.96)	0.03		0.82 (0.35, 1.89)	0.63	
Year cycle			0.62			0.89
2007–2012	0.60 (0.41,0.88)	0.01		0.72 (0.42,1.21)	0.20	
2013–2018	0.65 (0.33,1.28)	0.19		0.56 (0.32, 0.98)	0.04	
Vigorous/moderate recreational activities			0.36			0.20
Yes	0.80 (0.37, 1.72)	0.56		0.77 (0.42,1.41)	0.39	
No	0.53 (0.32,0.88)	0.02		0.52 (0.32, 0.85)	0.01	
Body Mass Index			0.51			0.83
Normal	0.65 (0.29,1.48)	0.30		0.78 (0.37, 1.62)	0.50	
Overweight	0.83 (0.45,1.55)	0.56		0.62 (0.32, 1.17)	0.14	
Obesity	0.52 (0.28,0.96)	0.04		0.64 (0.37,1.10)	0.10	
Educational status			0.87			0.89
>high school	0.59 (0.33,1.04)	0.07		0.67 (0.42,1.07)	0.09	
high school	0.72 (0.34,1.52)	0.38		0.62 (0.28, 1.35)	0.22	
<high school	0.67 (0.30,1.49)	0.32		0.79 (0.37, 1.67)	0.53	
Smoke			0.73			0.21
Now	0.55 (0.21, 1.47)	0.23		0.98 (0.40, 2.40)	0.96	
Former	0.82 (0.46,1.46)	0.50		0.40 (0.20, 0.79)	0.01	
Never	0.53 (0.30,0.93)	0.03		0.74 (0.41,1.34)	0.31	
Hypertension			0.15			0.94
Yes	0.75 (0.51,1.10)	0.14		0.69 (0.43,1.13)	0.14	
No	0.44 (0.20, 0.97)	0.04		0.67 (0.31, 1.44)	0.30	
Diabetes mellitus			0.30			0.14
Yes	0.51 (0.32,0.84)	0.01		0.40 (0.21,0.77)	0.01	
Borderline	0.45 (0.16, 1.30)	0.14		1.17 (0.53, 2.58)	0.70	
No	0.72 (0.39,1.33)	0.30		0.68 (0.35, 1.33)	0.26	

OR: odds ratio; CI, confidence interval. The model is adjusted for age, gender, race, BMI, FIR, education, smoking, physical activity, diabetes, hypertension, serum ldl-cholesterol, and energy intake. FIR, family income-to-poverty ratio.

## Data Availability

The data utilized to support the findings are available from the corresponding authors upon request.

## Supplementary Materials

The following supporting information can be downloaded at: https://www.mdpi.com/article/10.3390/nu14224908/s1, Figure S1: Flow chart of individuals with missing variables for each study year.
