# Supplementary figures and images for: Association of Dietary Live Microbe Intake with Cardiovascular Disease in US Adults: A Cross-Sectional Study of NHANES 2007–2018
Source: Nutrients. 2022 Nov 20;14(22):4908. doi: 10.3390/nu14224908 (PMC9698609; doi:10.3390/nu14224908)

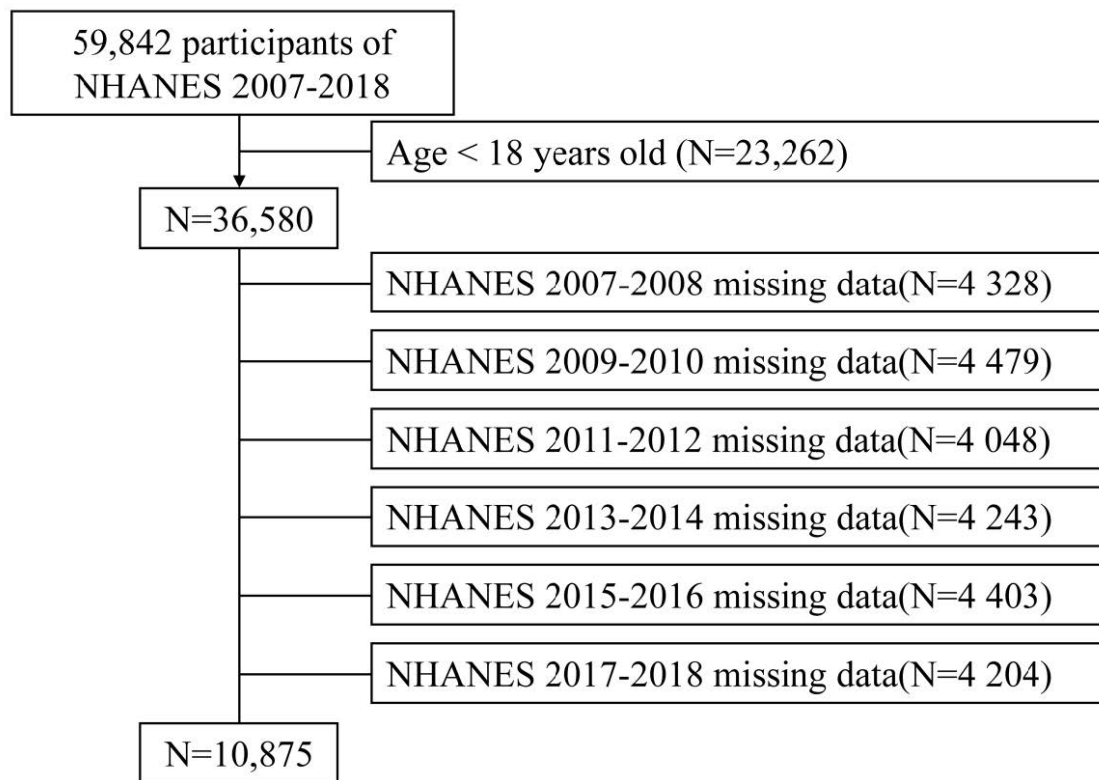

**Figure S1.** Flow chart of individuals with missing variables for each study year.

Supplement: Supplementary file 1 [file nutrients-14-04908-s001.zip › nutrients-2024164-supplementary.pdf]
